# Resistance and tolerance of ten carrot cultivars to the hawthorn-carrot aphid, *Dysaphis crataegi* Kalt., in Poland

**DOI:** 10.1371/journal.pone.0247978

**Published:** 2021-03-02

**Authors:** Maria Pobożniak, Małgorzata Gaborska, Tomasz Wójtowicz

**Affiliations:** 1 Department of Botany, Physiology and Plant Protection, Faculty of Biotechnology and Horticulture, University of Agriculture in Krakow, Krakow, Poland; 2 Department of Plant Breeding, Physiology and Seed Science, Faculty of Agriculture and Economics, University of Agriculture in Krakow, Krakow, Poland; University of Manitoba, CANADA

## Abstract

Damage caused to cultivated carrots by the hawthorn-carrot aphid, *Dysaphis crataegi* Kalt. (Hemiptera: Aphididae) is one of the factors limiting carrot production in Poland. Planting resistant and tolerant cultivars could reduce yield losses due to the damage caused by this pest. This study was conducted to evaluate the resistance and/or tolerance of 10 carrot genotypes to hawthorn-carrot aphid. Their field resistance was determined under field conditions based on five indicators, namely, mean number of alates (migrants) per plant and mean percentage of plants colonized by them, mean seasonal number of aphids per plant, mean number of aphids per plant and mean percentage of infested plants at peak abundance. Antibiosis experiments were conducted under laboratory conditions and pre-reproductive, reproductive time, fertility, and demographic parameters, represented by the net reproduction rate (*R*_*o*_), intrinsic rate of increase (*r*_*m*_) and mean generation time (*T*), were calculated. Five cultivars, Afro F_1_, Nipomo F_1_, Samba F_1_, White Satin F_1_, and Yellowstone showed field resistance. Antibiosis experiments revealed significant differences among the carrot cultivars in the length of the reproductive period, female fecundity in the time equal to the pre-reproduction time, and total progeny of hawthorn-carrot aphid. The intrinsic rate of natural increase (*r*_*m*_) for apterous aphids varied significantly, ranging between 0.181 (Nipomo F_1_) and 0.343 females/female/day (White Satin F_1_). Additionally, the estimated net reproductive rate (R_0_) was the lowest on Nipomo F_1_, and this genotype was determined to be resistant. Our results suggest that a very high density of trichomes on the leaf petioles (71.94 trichomes/cm^2^) could adversely affect the feeding, bionomy, and demographic parameters of hawthorn-carrot aphid on the cultivar Nipomo F_1_. In addition, Napa F_1_ and Kongo F_1_ demonstrated high tolerance. Considering all the results collectively, four genotypes, Afro F_1_, Kongo F_1_, Napa F_1_ and Nipomo F_1_, were relatively resistant/tolerant to the hawthorn-carrot aphid.

## Introduction

Poland contributes 15% of the total carrot production in the EU and is the largest producer of dried carrot [1]. The demand for high quality carrots, produced to the highest standards, is growing continually. These standards can be met by the application of Integrated Pest Management (IPM) principles. An important element of IPM is the cultivation of cultivars characterized by a higher degree of resistance and/or tolerance to the important pest species for the particular crop. Szwejda and Wrzodak [2] reported that due to the cultivation of carrots in Poland for decades, this plant has become a host for numerous species of harmful insects. Among the phytophagous species on carrot crops in Poland causing significant economic losses are carrot fly, *Psila rosae* Fabr., cutworms, *Agrotis* sp., and aphids. There are three species of aphids from Aphididae: willow-carrot aphid, *Cavariella aegopodii* Scop., carrot aphid, *Semiaphis dauci* Fabr., hawthorn-carrot aphid, *Dysaphis crataegi* Kalt. and one species from Pemphigidae: root aphid, *Pemphigus phenax* Börn. Et Blunck [2].

Currently, hawthorn-carrot aphid has been the most important herbivorous pest infesting carrot crops, besides carrot fly, in recent years in Poland [3–5]. It occurs in Europe, Central Asia, and North America, and includes a group of closely related subspecies. They all have hawthorn (*Crataegus* sp.) as the primary host. The secondary host depends on the subspecies involved. In Europe, hawthorn-carrot aphid migrates to wild and cultivated carrot [6]. In Poland, from the end of May or beginning of June until harvest, aphids create colonies at the base of leaf petioles, on the root neck, and on the roots [4, 7]. The same authors [4, 7] observed the development of 3 to 9 generations of hawthorn-carrot aphid on carrot per growing season. Dense sowing and a warm summer weather with a small amount of precipitation promote aphid development [7]. The negative effects of the hawthorn-carrot aphid are reflected in both the quantity and quality of the crop. For example, its feeding causes the reduction of carrot root mass, longitudinal cracking and greening of the base and upper parts of the roots. Goszczyński [8] showed adverse effects of its feeding on the photosynthesis and respiration of both primary and secondary hosts. In the roots of carrots damaged by hawthorn-carrot aphid, there were decreases in the dry weight, sugars, and β-carotene and increases in the nitrogen and protein contents [9].

The cultivation of resistant or tolerant cultivars is a viable alternative to the use of chemical methods to manage many crop pests. Resistant cultivars have physical and/or chemical defense mechanisms that protect them against pest infestation and feeding [10, 11]. Tolerant cultivars have high regeneration and damage compensation abilities, so that they can produce higher yields than susceptible cultivars [10, 12]. Cultivars with even partial resistance are desirable in production systems, and their cultivation is considered to be the best method of pest management [13].

Plant resistance can broadly be classified as antixenosis, meaning nonpreference, or as antibiosis, meaning how suitable a plant is for a herbivore [14–16]. It has been demonstrated that these mechanisms of plant resistance can be inferred from the behavior of aphids [17, 18]. These plant-pest interactions refer to two different phenomena, namely the search for the plant and its acceptance (antixenosis), and the impact of the plant as food on the biology, fecundity, and health of the pest (antibiosis) [15, 19, 20]. Antixenosis is based on behavioral avoidance of a host due to a feature or set of features that deter insects from settling and feeding [14]. The expression of antixenosis in genotypes may be a consequence of both chemical and morphological features in plant and mainly affects the visual and olfactory stimuli involved in the host-finding behavior of aphids [11, 21, 22]. In contrast, antibiosis refers to adverse effects on the biology of insects and their progeny (survival, development, and reproduction) as a result of feeding on resistant plant genotypes that may contain secondary plant compounds and/or be of poor nutritional quality [23, 24].

The measurement of insect population size in the field under environmental conditions is used by entomologists as tools for the first stage of selection of resistant plant material. The existence of plant resistance can indicate that the plant possesses a mechanism for antixenosis and/or antibiosis. They are not the only determinants of aphid development and survival on crops in the field, because there are also biotic factors (predators, parasitoids) and abiotic factors (temperature, wind, precipitation, soil) [25, 26].

Antixenosis is an important component of resistance because it reduces the initial infestation levels, however, in monoculture, this mechanism may be broken down in the absence of the preferred host plant. In this case, the pests may eventually accept a less favored host [27, 28]. Antixenosis and antibiosis are often correlated because many colonizing adult herbivores choose the plants that are suitable for their offspring [29, 30]. However, aphids may not always be able to make optimal host plant choices because their flight is strongly affected by environmental factors such as wind speed, direction, and temperature [31]. Furthermore, for rapidly developing herbivores such as aphids, in which a number of generations can develop on the host plant during one season, antibiosis becomes increasingly important as time passes, while the initial choice of the colonizing herbivores will become less significant [16].

Plant tolerance to insect pests has been described as a unique category of resistance because tolerance does not interfere with pest insect physiology and behavior, as observed in antibiotic or antixenotic resistance [12, 32]. Tolerance indicates the ability of a host plant to withstand or recover from herbivore damage through compensatory physiological processes and growth [33]. Tolerance may be evidenced by increased net photosynthetic rate after damage [34] or up-regulation of detoxification mechanisms to counteract the harmful effects of aphids [35]. Painter [15] included tolerance in the concept of resistance; however, it was later allocated its own category [36].

The only varieties of carrot resistant to the carrot fly were developed by Ellis [37]. The first report of resistance to carrot fly was from the late nineteenth century but research intensified worldwide in the 1970s. This body of research resulted in the identification of several Nantes carrot varieties with partial resistance attributable to antibiosis mechanisms which correlated with the concentration of chlorogenic acid in the roots [38].

A better understanding of the resistance of carrot genotypes to the hawthorn-carrot aphid is essential for carrot breeders to improve the resistance or tolerance of carrot cultivars to this pest. However, to date no studies have investigated carrot resistance to the hawthorn-carrot aphid. Therefore, this research was conducted to identify carrot genotypes resistant to the colonization (antixenosis) and development (antibiosis) of the hawthorn-carrot aphid under both field and laboratory conditions, including the determination of tolerance to feeding.

## Materials and methods

All animal work was conducted according to relevant national and international guidelines. Insect collection permits were not required since the area where the aphids were collected did not contain any strictly protected areas, and hawthorn-carrot aphid is not under protection in Europe. Also no permits were required to use the hawthorn carrot aphid for experiment due to the observational nature of the data collection. Formal approval for the experiment was obtained from University of Agriculture in Krakow.

All of the carrot cultivars used in the study are commercially available and were obtained from Polish companies, namely, Polan in Krakow (Rumba F_1_, Samba F_1_ with an orange roots); PlantiCo in Gołębiew (Afro F_1_, Kongo F_1_ with orange roots), and Bejo Zaden Poland in Ożarów Mazowiecki (Kazan F_1_, Napa F_1_, Nipomo F_1_ with orange roots, and Deep Purple F_1_ with purple roots, Yellowstone with a yellow roots, and White Satin F_1_ with white roots). Earlier preliminary screening of large number of new and F_1_ hybrid carrot genotypes indicated that ten selected genotypes might possess resistance or tolerance to the hawthorn-carrot aphid (unpublished data).

Field experiments were conducted at the Experimental Station of the University of Agriculture in Krakow which is located in Mydlniki (near Krakow, in southern Poland, at 50°04′N, 19°51′E and, 207 m above sea level) on a typical brown soil with a pH of 6·5 and an organic carbon content of 18 g/kg. The trial was established in a completely randomized design with three replications for each of the carrot cultivars. On an area of 49 m × 9.8 m, 10 plots were set up with each containing 3 rows. The plots, measuring 10.4 m^2^ (4 m × 2.6 m), were separated by 1-m-wide paths. The plots were also separated from the neighboring crops (onion and red beets) by a 1-m path. Seeds were sown at the rate of 3.5 kg/ha in rows, 0.3 m apart on April 19, 2011 and April 24, 2012. Plant density was approximately 150 plants/m^2^. Fertilization was in line with integrated production recommendations. No chemical treatments were applied, and weeds from plots and paths were removed mechanically and manually.

Meteorological data (air temperature and rainfall) were recorded with a HOBO water temperature Pro data logger (Onset Computer Corp., Bourne, USA) at hourly intervals at the trial site from May to September in 2011 and 2012 (S1 Table).

### Field resistance experiment

The identification of resistance to host plant infestation (selection of plants for settlement) and determination of abundance of the hawthorn-carrot aphid were carried out under field conditions. For this purpose, from the end of May until harvest (end of September), 30 plants of each tested cultivar (10 × 3 replications) were taken from plots, on average every 7–10 days, and analyzed in the laboratory under a stereoscopic microscope. Each time, aphids feeding on the leaf petioles and root necks, as well as those present on the underground parts of the carrots, were counted and identified to the species level. The taxonomic identification was made on the basis of keys developed by Müller [6] and Cichocka [39].

In the assessment of the level of field resistance assigned cultivars to plant colonization by migrants, two indicators were used, the mean number of alates (migrants) per plant and the mean percentage of plants colonized by them. They were determined on the basis of data collected during the migration period, which lasted from May 31 to June 29, 2011, and from May 30 to 27 June, 2012. To estimate the level of field resistance from the number of feeding aphids (alates and apterous), three indicators were used: mean seasonal number of aphids per plant, mean number of aphids per plant at peak abundance, and mean percentage of infested plants at peak abundance. The indicators were assigned a value from 1 to 4. The highest numbers of points were ascribed to the lowest values for the indicators, which in turn indicated the highest level of cultivar resistance. Because the abundance of the aphids populations differed significantly in 2011 and 2012, different scales were used for the indicators in each year (Table 1).

**Table 1:** The number of points assigned to indicators of the field resistance of carrot cultivars to the hawthorn carrot aphid, *Dysaphis crataegi*.

Indicators	Year	Scale (number of points)			
		4	3	2	1
Mean number of alate aphids per plant	2011	0.00–0.02	0.03–0.05	0.06–0.15	>0.15
	2012	0.00–0.20	0.21–0.40	0.41–0.60	>0.60
Mean percentage of plants colonized by alate aphids	2011	0.00–0.50	0.51–1.00	1.01–3.00	>3.00
	2012	0.00–4.50	4.51–6.00	6.01–11.00	>11.00
Mean seasonal number of aphids per plant	2011	0.00–0.05	0.06–0.50	0.51–1.00	>1.00
	2012	0.00–1.00	1.01–3.00	3.01–8.00	>8.00
Mean number of aphids per plant at peak abundance	2011	0.00–0.25	0.26–3.00	3.01–7.00	>7.00
	2012	0.00–5.00	5.01–15.00	15.01–25.00	>25.00
Mean percentage of infested plants at peak aphid abundance	2011	0.00–1.00	1.01–10.00	10.01–20.00	>20.00
	2012	0.00–10.00	10.01–25.00	25.01–50.00	>50.00

The final level of field resistance was based on the average number of points from two years, assigned separately for plant colonization by migrants, and the number of feeding aphids and percentage of populated plants. Four levels of field resistance were used to classify the carrot genotypes: resistant—with a high degree of field resistance (>3.50 points), moderately resistant—with moderate degree of field resistance (3.50–2.51 points), susceptible—with low degree of field resistance (2.50–1.5 points), and highly susceptible—with very low degree of field resistance (<1.5 points).

### Morphological and chemical analyses of plant material

The plant materials used to conduct morphological and chemical analyses were collected in the last week of June 2011. The length and thickness of trichomes on the leaves and leaf petioles of three leaves collected from each replication (plot) of the carrot cultivars were determined under a stereo-microscope. The surface of each leaf and leaf petiole was scanned with the use of the Multi Scan Base Software Program (Computer Scanning System, Warsaw, Poland) to determine the density of trichomes per cm^2^. The contents of soluble sugars in the leaf petioles and root neck apexes of each cultivar were determined by using the anthrone test [40], and for the determination content of reducing sugars, the hexacyanoferrate method described by Nath and Singh [41] was used. In addition, the sucrose concentration was calculated as the difference between the concentrations of total soluble sugars and reducing sugars (total sugars–reducing sugars ≈ sucrose).

### Antibiosis study

For the antibiosis study, aphid samples were collected from carrot plants growing in the field on June 29, 2012. After collection, wingless aphids, along with pieces of the host plants, were transferred to the laboratory. Aphids were then reared for two generations, on plants of the same 10 carrot cultivars as used in the field experiment in an air-conditioned room at 21 ± 2°C, 65 ± 5% relative humidity (RH), and with a 16:8 (L:D) h photoperiod. In the experiment, the same 10 tested cultivars of carrot were used. Five seeds of each cultivar were sown in plastic pots that were 10 cm deep and 7 cm in diameter. The plants were grown in a standard substrate in an air-conditioned growing room at 21 ± 2°C, 65 ± 5% relative humidity (RH) and with a 16:8 (L:D) h photoperiod. All plants were watered regularly with tap water only. Seven weeks after their emergence all plants, except the best grown one, were removed.

All bioassays were undertaken in a growth chamber at 21 ± 2°C, 80 ± 5% RH, and 16:8 (L:D) h photoperiod. One adult wingless female was placed on a leaf petiole near the root neck of each carrot plant of each cultivar at age 7–8 weeks with the use of a brush. On the following day, the plants were thoroughly examined and only one nymph was allowed to remain on each plant after the removal of the other nymphs and female aphids. Ten replicates were established and analyzed for each cultivar, this number of replicates is sufficient for this type of research [42]. The experiment was begun with one first nymphal instar per plant, and the total number of nymphs produced daily was counted. These nymphs were removed after counting from the plants, and this process continued until all of the aphids had died. In the life table, the bionomic parameters, pre-reproduction period (*d*), reproduction period and mean number of nymphs produced, were calculated.

The demographic parameters of the hawthorn-carrot aphid, including the net reproduction rate (R_o_), the intrinsic rate of increase (r_m_), and the mean generation time (*T*), were calculated by using the method proposed by Birch [43] and Wyatt and White [44], in which:

$$r_{m}=\frac{0.738∙(ln\;M_{d})}{d}$$

$$R_{o}=e^{r_{m}∙T}$$

$$T=\frac{d}{0.738}$$

In this method, *d* is the time period before nymph production, *M*_*d*_ is the number of progeny in the time equal to *d*, and 0.738 is the correction constant.

Four degrees of antibiosis for aphid development were determined from the intrinsic rate of increase (r_m_). The calculated values of r_m_ were assigned from 1 to 4 points according to a scale based on the value of r_m_ and the result of the paired-bootstrap test (Table 5). A higher number of points was given to lower values of r_m_, which indicated a higher level of antibiosis. The final level of plant resistance was determined in the same way as for field resistance.

### Tolerance experiment

Tolerance screening was carried out in a field experiment in the period 2011–2012. In both years, two treatments were used for each tested cultivar: C–control treatment (with uninfested plants) and D–treatment with plants colonized by hawthorn-carrot aphid. Each treatment consisted of 3 enclosures (repetitions). After the carrot plants’ emergence, all plants in the middle rows were covered with transparent muslin crop covers to protect them against insect infestation. In both seasons, in the second half of June, the crop covers were removed, and metal constructions (frames) 50 cm high and 50 cm wide and covered with thin, transparent gauze, were installed on selected plants. Eight carrot plants were grown under each enclosure. On June 29, 2011 and June 28, 2012, the eight isolated plants in the D treatment were infested with young, wingless hawthorn-carrot aphid females, with 8 specimens added to each enclosure and specifically, one female per plant. The females had been collected from the experimental plots containing the same cultivar. After 6 weeks, the enclosures were removed, and were again covered separately with muslin to protect them against another aphid infestation. While the plants were growing in their enclosures, the aphids were not counted on them to avoid aphid spread and colony destruction.

During the carrot harvest period in September, for each treatment and cultivar, the length (in cm) and weight (in g) of each carrot root were determined. Following those procedures, the contents of sugars and carotenoids in the roots of the carrots of both treatments in the experiment, C and D, were determined. The contents of soluble and reducing sugars in the carrot roots was determined by the same method as for the leaf petioles and the root neck apexes. The concentrations of carotenoids was determined on a JASCO V-530 spectrophotometer; the sum of carotenoids concentrations was converted into β-carotene, by using its absorption coefficient of 250 at 450 nm [45]. In addition, the sucrose concentration was calculated as the difference between total soluble sugars and reducing sugars (total sugars–reducing sugars ≈ sucrose).

Based on the significant or insignificant impacts of aphid feeding (increase or decrease compared to control plants) on root length, weight and contents of reducing sugars, sucrose, and carotenoids, scores from 1 to 4 points were assigned to each cultivar. The final level of tolerance was based on the average number of points from two years. Four levels of tolerance were used to classify the carrot genotypes: very low (≤2.50 points), low (2.60–2.70 points), moderate (2.80–2.90 points), and high (≥3.00 points) tolerance.

### Data analysis

Statistical analyses were performed with Statistica 13 software (Dell Inc. 2016). For all ANOVA analyses residual plots were checked for normality of residuals. In the case of the absence of normality, the data were normalized with log_10_(x+1) transformation. The tables and figures contain untransformed data.

The interrelationship between two variables, colonization of carrot plant (non-colonized/colonized) and carrot cultivar (10 cultivars) in terms of the number of infested plants during the aphid migration period and at peak aphid abundance, were analyzed in 2×10 contingency tables with the χ^2^ test (P<0.05). One-way ANOVA (the factor was carrot cultivar) was performed on the mean number of alate (migrant) aphids per plant throughout the migration period, mean seasonal number of aphids per plant, mean number of aphids per plant at peak abundance, trichome measurements (length, thickness, and density), and the contents of soluble and reducing sugars in the leaf petioles and root neck apexes. The differentiation of the values of means was determined with the Duncan’s Multiple Range Test (P<0.05).

Two-way ANOVA was performed for cultivars and treatments (controls/colonized plants) nested within cultivars for the tolerance data, i.e., the length and weight of the root and the contents of sugars and carotenoids in the roots. When significant differences between treatment means were detected with ANOVA, within each cultivar the control mean and mean obtained for colonized plants were compared with Student’s t test (P<0.05). The difference between the control and the colonized plants for the tolerance parameters was recalculated as a percentage of the control (Fig 5).

To examine the relationship between the indicators of field resistance, demographic parameters (*R*_*o*_, *T*) and the density of trichomes on the leaves and leaf petioles of the tested carrots cultivars, the Pearson’s correlation coefficient (*r*) was calculated, and significance was set at P<0.05.

The demographic parameters of hawthorn-carrot aphid were analyzed using the bootstrap procedure with 5,000 bootstraps and one-way ANOVA [46]. The differences between the cultivars were compared with the paired bootstrap test (P<0.05).

To examine the linkage between the cultivars in terms of resistance mechanisms, Ward’s hierarchical clustering was applied [47]. The dendrogram for field resistance was generated based on the data from 2011 and 2012; specifically, mean number of alates (migrants) per plant, seasonal mean number of aphids per plant, mean number of aphids per plant at peak abundance, mean percentage of colonized plants during aphid migration, and mean percentage of colonized plants at peak abundance. The dendrogram for antibiosis was generated from the bionomic and demographic data of the hawthorn-carrot aphid obtained under laboratory conditions. The dendrograms were created to provide a comprehensive perspective of the data for the mechanisms of resistance and were helpful during the final assessment of the tested cultivars.

## Results

### Field resistance assessment

**Fig 1:**
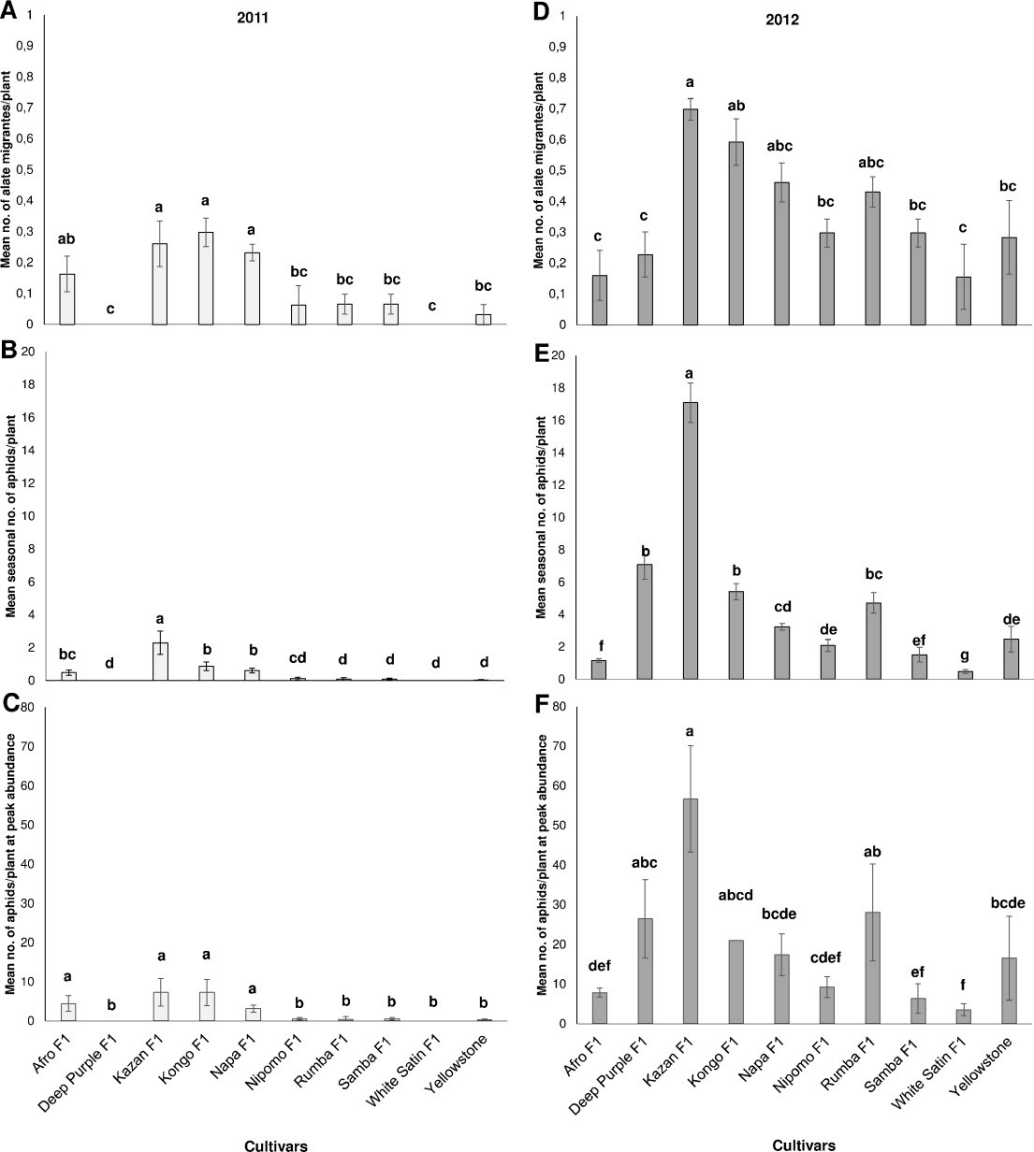
Mean *Dysaphis crataegi* abundance on carrot genotypes: (A, D) during the aphid migration period), (B, E) throughout the growing season and (C, F) at peak abundance. Means with the same letters on each bar are not significantly different (Duncan’s Multiple Range Test P< 0.05.

**Fig 2:**
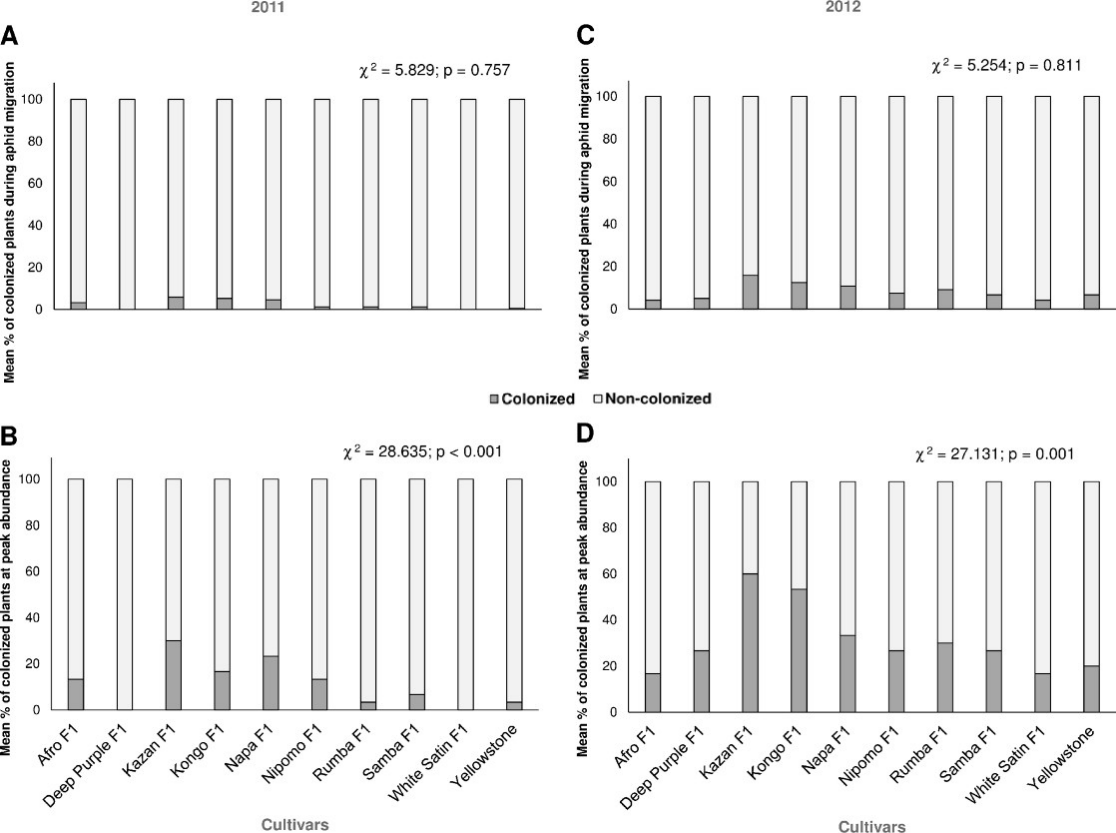
Colonization of carrot plants by *Dysaphis crataegi*: (A, C) during aphid migration and (B, D) at peak aphid abundance (Chi-square (χ^2^) test, df = 9).

Field studies showed that the number of migrant alates of the hawthorn carrot aphid, as well as the colony size and percentage of colonized plants, were influenced by carrot genotype and year. Aphid population sizes were higher in 2012 than in 2011, when the May, June (excluding the first 10 days), July and August average temperatures were higher and the total rainfall was lower (Figs 1A–1F and 2A–2D, S1 Table).

In the 2011 season, the hawthorn-carrot aphid was found on eight carrot cultivars (it was not recorded on Deep Purple F_1_ and White Satin F_1_) (Fig 1A and 1B). Significantly more migrants infested cv. Kazan F_1_, Kongo F_1_ and Napa F_1_ than the other infested carrot genotypes with the exception of only Afro F_1_ (F = 5.417; df = 9, 20; P<0.001) (Fig 1A). The percentage of carrot plants colonized by alates was not significantly affected by the cultivar, and no more than 6.0% of infested plants were found (Fig 2A).

A significantly higher mean seasonal number of aphids was detected on Kazan F_1_ than on the other cultivars (F = 16.584; df = 9, 20; P<0.001). In addition, the mean seasonal number of aphids on Kongo F_1_ significantly differed from the other tested cultivars, with the exception of only Afro F_1_ and Napa F_1_. Cultivar Yellowstone was infested with the lowest number of aphids, followed by Rumba F_1_, Nipomo F_1_, and Samba F_1_ (Fig 1B).

For the mean number of aphids at peak abundance the Duncan’s test produced two homogenous groups from the 10 carrot cultivars, four were aphid-susceptible: Afro F_1_, Kazan F_1_, Kongo F_1_, and Napa F_1_; and in the second group, the six genotypes were resistant: Deep Purple F_1_, Nipomo F_1_, Rumba F_1_, Samba F_1_, White Satin F_1_, and Yellowstone (F = 10.540; df = 9, 20; P<0.001) (Fig 1C). The percentage of infested carrot plants at peak aphid abundance was significantly affected by the cultivar. The percentage of infested plants showed the highest differences between cultivars in terms of susceptibility to hawthorn-carrot aphid; Kazan F_1_ and Napa F_1_ attracted the most aphids, Rumba F_1_ and Yellowstone were the least infested, and Deep Purple F_1_ and White Satin F_1_ were not infested (Fig 2A).

In contrast, all of the tested cultivars were infested by hawthorn-carrot aphid in 2012 (Fig 1D and 1E). The migration of winged females and the aphid populations were much higher and provided much more representative data than in the previous year (Fig 1A and 1D).

The highest number of migrating alates was found on cv. Kazan F_1_ in comparison to the others cultivars, with the exception of cv. Kongo F_1_, Napa F_1_ and Rumba F_1_ (F = 3.351; df = 9; 20, P<0.001). White Satin F_1_, Afro F_1_ and Deep Purple F_1_ were colonized by the lowest number of winged females (Fig 1D). However, the percentage of plants colonized by migrating aphids was not significantly dependent on the carrot cultivar (Fig 2C).

In 2012, aphid abundance throughout the growing season was significantly higher on the cv. Kazan F_1_ (F = 40,419; df = 9, 20; P<0.001) (Fig 1E). Deep Purple F_1_ and Kongo F_1_ fell into the second homogenous group when Duncan’s test was performed, while aphid population growth was significantly lower on Afro F_1_, Napa F_1_, Nipomo F_1_, Samba F_1_, White Satin F_1_, and Yellowstone (Fig 1E). Additionally, a significant difference was found at peak aphid abundance between Kazan F_1_ and the other tested cultivars except Deep Purple F_1_, Kongo F_1_, and Rumba F_1_ (F = 5.990; df = 9, 20; P<0.001) (Fig 1F). In 2012, the highest mean number of aphids at peak abundance was on Kazan F_1_, and the value exceeded by 3-, 3-, 6-, 7-, 9-, and, 15-fold the mean number of individuals at peak abundance on the 6 least susceptible cultivars, namely, Napa F_1_, Yellowstone, Nipomo F_1_, Afro F_1_, Samba F_1_, and White Satin F_1_, respectively (Fig 1F). Overall, there was a significant relationship between the percentage of carrot plants colonized by the hawthorn-carrot aphid at the peak aphid abundance and genotype. Aphid settling reduced on the above-mentioned 6 cultivars was below 30%, while the most susceptible Kazan F_1_ was inhabited by 62% (Fig 2D).

**Fig 3:**
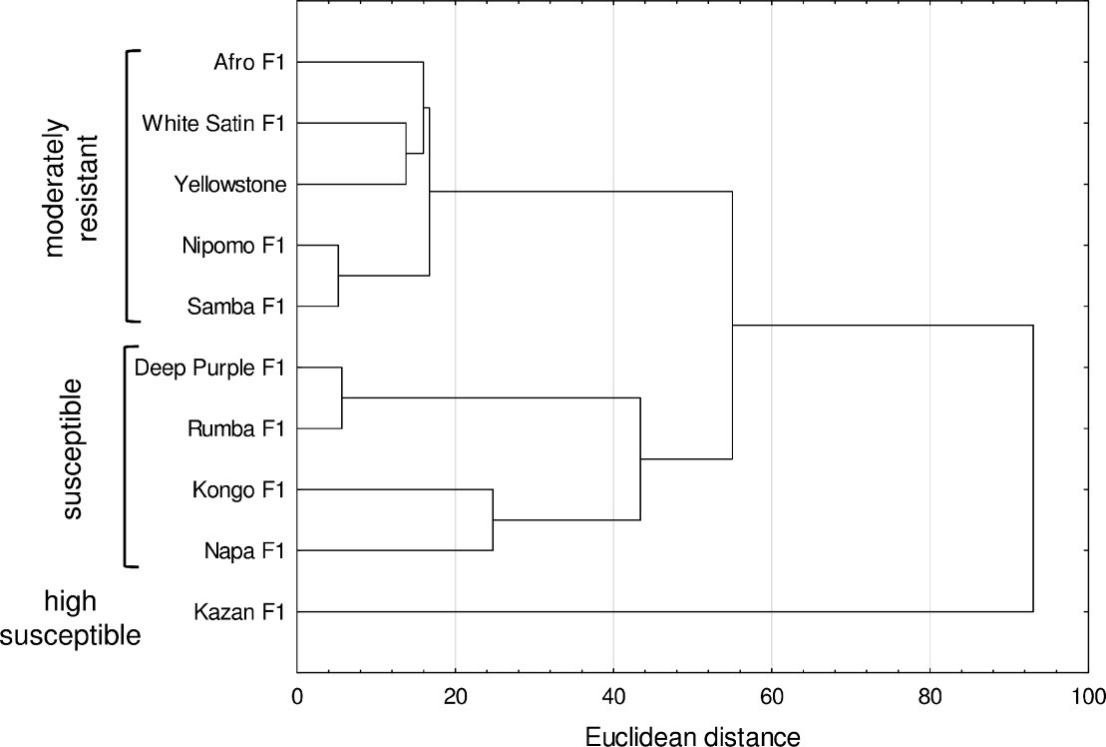
Dendrogram showing the clustering of carrot cultivars based on data obtained from field assessments: Mean number of alates (migrants), mean seasonal number of aphids per plant, mean number of aphids per plant at peak abundance, mean percentage of colonized plants during aphid migration, and mean percentage of colonized plants at peak aphid abundance.

A dendrogram based on data obtained from the field assessment showed two distinct groups of cultivars (Fig 3). In the first cluster there were 9 cultivars, which formed 2 subgroups. In the first subgroup are the cultivars with moderate resistance, of which Nipomo F_1_ and Samba F_1_ had the greatest similarity in terms of plant colonization and number of feeding aphids. The second subgroup included cultivars with greater susceptibility, which reflected higher numbers of migrating aphids and percentages of colonized plants or the number of foraging aphids. The second cluster included cv. Kazan F_1_ with the lowest field resistance, which had especially in 2012, the highest number of feeding aphids and populated plants (Fig 3).

Based on these results, five genotypes with field resistance were identified: Afro F_1_, Nipomo F_1_, Samba F_1_, White Satin F_1_, and Yellowstone, and two highly susceptible genotypes, Kazan F_1_ and Kongo F_1_ (Table 2). These two cultivars were the most attractive to hawthorn-carrot aphid for colonization; with Kazan F_1_ better supporting the development of aphids under field conditions. Specifically, on Kazan F_1_ at peak aphid abundance there was a mean of 56.7 aphids per plant, which was more than 2.5 times the number on Kongo F_1_ (21.0 aphids per plant) in 2012 (Fig 1F). In addition, Deep Purple F_1_, Napa F_1_ and Rumba F_1_ were classified as susceptible to the hawthorn-carrot aphid (Table 2).

**Table 2:** Levels of resistance and tolerance of ten carrot cultivars to the hawthorn-carrot aphid, *Dysaphis crataegi*.

Cultivar	Resistance						Tolerance	
	Field resistance				Antibiosis			
	Plant colonization by migrants		No. of feeding aphids and populated plants					
	No. of points	Final level	No. of points	Final level	No. of points	Final level	No. of points	Final level
Afro F_1_	2.5	susceptible	2.66	moderately resistant	3	moderately resistant	2.4	very low
Deep Purple F_1_	3.50	moderately resistant	2.83	susceptible*	2	susceptible	2.5	very low
Kazan F_1_	1.00	high susceptible	1.00	high susceptible	2	susceptible	2.7	low
Kongo F_1_	1.25	high susceptible	1.66	susceptible	2	susceptible	3.0	high
Napa F_1_	1.5	susceptible	1.83	susceptible	2	susceptible	3.2	high
Nipomo F_1_	2.25	susceptible	2.83	moderately resistant	4	resistant	2.8	moderate
Rumba F_1_	2.0	susceptible	2.33	susceptible	2	susceptible	2.9	moderate
Samba F_1_	2.5	susceptible	2.83	moderately resistant	1	high susceptible	2.5	very low
White Satin F_1_	4.0	resistant	3.83	moderately resistant	1	high susceptible	2.9	moderate
Yellowstone	2.75	moderately resistant	3.00	moderately resistant	2	susceptible	2.4	very low

* The abundance of *D*. *crataegi* on cv. Deep Purple F_1_ in the more representative growing season in 2012 was high, which meant that the scores for field resistance allocated for the number of feeding aphids and percentage of populated plants were low. Therefore it was classified as susceptible in the final assessment.

There were significant differences among the studied carrot genotypes with respect to the length, thickness, and density of leaf and leaf petiole trichomes (Table 3). The longest trichomes were on Deep Purple F_1_ but the thickest were on Nipomo F_1_. Conversely, the shortest and the thinnest trichomes were on White Satin F_1_ and Yellowstone. The highest numbers of trichomes were found on the leaves of Deep Purple F_1_ but the highest densities were on the leaf petioles of Nipomo F_1_. In contrast, the lowest trichome densities were observed on White Satin F_1_ and Yellowstone leaves, and no trichomes were found on White Satin F_1_ leaf petioles (Table 3). Despite these differences, no significant correlation was found between the field resistance indicators and the density of trichomes on the leaves and leaf petioles of the tested carrot cultivars (S2 Table).

**Table 3:** Characteristics of leaf and leaf petiole trichomes of 10 carrot genotypes (one-way ANOVA, factor df = 9, error df = 20).

Cultivar	Mean length of leaf trichomes (± SE) (μm)	Mean thickness of leaf trichomes (± SE) (μm)	Density of trichomes	
			Mean number of trichomes /1 cm^2^ of leaf (± SE)	Mean number of trichomes/1 cm^2^ of leaf petiole (± SE)
Afro F_1_	313.33 ± 8.82 d	62.33 ± 2.03 c	21.61 ± 1.65 e	16.34 ± 0.55 e
Deep Purple F_1_	476.66 ± 6.66 a	66.00 ± 1.15 bc	57.03 ± 1.07 a	9.85 ± 0.09 f
Kazan F_1_	356.66 ± 3.33 bc	62.67 ± 0.33 c	31.13 ± 1.13 d	14.75 ± 1.12 ef
Kongo F_1_	266.66 ± 8.18 f	62.00 ± 0.78 c	24.53 ± 0.61 e	2.66 ± 0.29 g
Napa F_1_	296.66 ± 3.33 de	63.67 ± 0.88 bc	44.82 ± 0.06 b	31.51 ±20.19 d
Nipomo F_1_	363.33 ± 3.33 bc	72.00 ± 1.15 a	43.69 ± 1.84 b	71.94 ± 4.47 a
Samba F_1_	343.33 ± 3.33 c	66.00 ± 1.15 bc	14.10 ± 0.61 f	41.09 ± 1.12 c
Rumba F_1_	376.66 ± 14.53 b	69.00 ± 2.30 ab	35.16± 0.72 c	57.58 ± 0.52 b
White Satin F_1_	270.00 ± 17.32 ef	50.00 ± 3.05 e	14.11 ± 1.69 f	0.00 ± 0.00 g
Yellowstone	216.66 ± 6.66 g	56.00 ± 0.58 d	3.85 ± 0.09 g	2.20 ± 0.11 g
F-value	63.320	13.003	215.154	222.421
P-value	<0.001	<0.001	<0.001	<0.001

Means within a column followed by the same letter(s) are not significantly different (Duncan’s Multiple Range Test P<0.05).

The 10 tested carrot genotypes had different sugar contents in the leaf petioles and the root neck apexes (Table 4). The cultivars, White Satin F_1_ and Yellowstone, had significantly higher concentrations of soluble sugars and sucrose than the other eight cultivars (Table 4) and high concentrations were also found in Napa F_1_. In the other cultivars, the contents of soluble sugars and sucrose did not differ significantly and were the lowest in Afro F_1_. The highest concentrations of reducing sugars were found in the leaf petioles and root neck apexes of the genotype Nipomo F_1_ and the lowest were in Napa F_1_ (Table 4).

**Table 4:** The contents of sugars in the leaf petiole and root neck apex of the tested carrot genotypes (one-way ANOVA, factor df = 9, error df = 20).

Cultivar	Mean quantity (± SE) [mg /100 g FW*]		
	Soluble sugars	Sucrose	Reducing sugars
Afro F_1_	3.91 ± 0.07 c	2.55 ± 0.01 c	1.36 ± 0.07 bcd
Deep Purple F_1_	4.53 ± 0.15 c	3.26 ± 0.16 c	1.27 ± 0.04 cd
Kazan F_1_	4.64 ± 0.02 c	3.12 ± 0.02 c	1.52 ± 0.04 ab
Kongo F_1_	4.20 ± 0.21 c	2.78 ± 0.18 c	1.42 ± 0.04 abc
Napa F_1_	6.06 ± 0.48 b	4.85 ± 0.46 b	1.21 ± 0.03 d
Nipomo F_1_	4.34 ± 0.38 c	2.80 ± 0.48 c	1.53 ± 0.11 a
Rumba F_1_	4.35 ± 0.45 c	2.83 ± 0.46 c	1.51 ± 0.02 ab
Samba F_1_	4.61 ± 0.07 c	3.11 ± 0.05 c	1.49 ± 0.02 ab
White Satin F_1_	8.33 ± 0.47 a	7.03 ± 0.4 3a	1.29 ± 0.03 cd
Yellowstone	7.6± 0.04 a	6.34 ± 0.06 a	1.31 ± 0.02 cd
F-value	26.736	28.661	5.755
P-value	<0.001	<0.001	<0.001

* FW = Fresh weight.

Means within a column followed by the same letter(s) are not significantly different (Duncan’s Multiple Range Test P<0.05).

### Antibiosis assessment

#### Bionomic parameters

The investigation of hawthorn-carrot aphid population growth clearly demonstrated that the tested carrot genotypes differ in their suitability as host plants. The results indicate a high level of antibiosis in the cultivar Nipomo F_1_ and a moderate level in Afro F_1_, but very low levels in the others eight cultivars. The White Satin F_1_ genotype provideed the most favorable conditions for aphid development under laboratory conditions (Table 5).

**Table 5:** Bionomic and demographic data for *Dysaphis crataegi* (mean ± SE) on ten carrot cultivars.

Cultivars	Pre-reproductive period (*d*) (day)	Reproductive period (day)	Fecundity of females *M*_*d*_ (nymphs/female)	Total progeny (nymphs/female)	*r*_*m*_ (females/female/day)	*T* (day)	*R*_*o*_ (females/female/generation)
Afro F_1_	6.3±0.30 a	5.4±0.62 d	8.3±0.40 b	8.5±0.5 e	0.253±0.014 c	8.5±0.41 a	8.3±0.44 c
Deep Purple F_1_	6.2±0.20 a	7.4±0.48 b	11.4±0.72 a	12.4±0.6 cd	0.290±0.012 b	8.4±0.27 a	11.4±0.58 ab
Kazan F_1_	5.9±0.23 a	7.4±0.64 b	11.0±0.87 a	15.1±1.3 ab	0.300±0.016 ab	8.0±0.32 a	11.0±0.80 ab
Kongo F_1_	6.1±0.23 a	7.6±0.64 b	13.1±1.03 a	16.1±1.7 ab	0.314±0.018 ab	8.3±0.32 a	13.1±0.71 a
Napa F_1_	5.9±0.18 a	8.6±0.58 ab	12.9±0.89 a	18.1±1.0 a	0.321±0.015 ab	8.0±0.24 a	12.9±0.68 a
Nipomo F_1_	6.0±0.26 a	3.5±0.48 e	4.4±0.48 c	4.6±0.3 f	0.181±0.021 d	8.1±0.35 a	4.4±1.01 d
RumbaF_1_	5.6±0.27 a	5.8±0.63 cd	10.0±1.01 ab	11.8±1.1 d	0.304±0.020 b	7.6±0.36 a	10.0±0.95 bc
SambaF_1_	5.7±0.30 a	6.0±0.58 cd	12.6±0.92 a	15.3±0.9 ab	0.333±0.021 ab	7.7±0.41 a	12.6±0.74 a
White Satin F_1_	5.6±0.31 a	9.2±0.85 a	13.0±0.92 a	17.4±1.2 a	0.343±0.018 a	7.6±0.41 a	13.0±0.67 a
Yellowstone	6.0±0.33 a	8.2±0.49 ab	12.0±1.94 a	14.6±0.6 b	0.301±0.019 ab	8.1±0.45 a	12.0±1.31 ab

*d* prereproduction time is the time from birth to the first production of nymphs; *M*_*d*_ the number of nymphs produced in the time equal to *d; r*_*m*_: intrinsic rate of population growth; *R*_*o*_: net reproductive rate; *T*: mean generation time.

Means within the same column followed by the same letters are not significantly different (Paired-bootstrap test at 5% significance level).

#### Demographic parameters

The calculated intrinsic rate of increase (*r*_*m*_*)* of the hawthorn-carrot aphid on the ten tested carrot cultivars ranged between 0.181 and 0.343 females/female/ day. The *r*_*m*_ value for hawthorn-carrot aphid on White Satin F_1_ was significantly higher than for Deep Purple F_1_, Afro F_1_, and Nipomo F_1_, and these values also differed significantly from each other (Table 5). However, the effect of cultivar on the mean generation time (*T*) was not significant. Table 5 also shows the values for the net reproductive rate (R_o_ (females/female/generation)) of the aphids on the ten genotypes. The values for this parameter indicated that the aphid has a high reproductive capacity. The *R*_*o*_ values for Kongo F_1_, White Satin F_1_, Napa F_1_, and Rumba F_1_ were significantly higher than that on Afro F_1_ and Nipomo F_1_ (Table 5).

**Fig 4:**
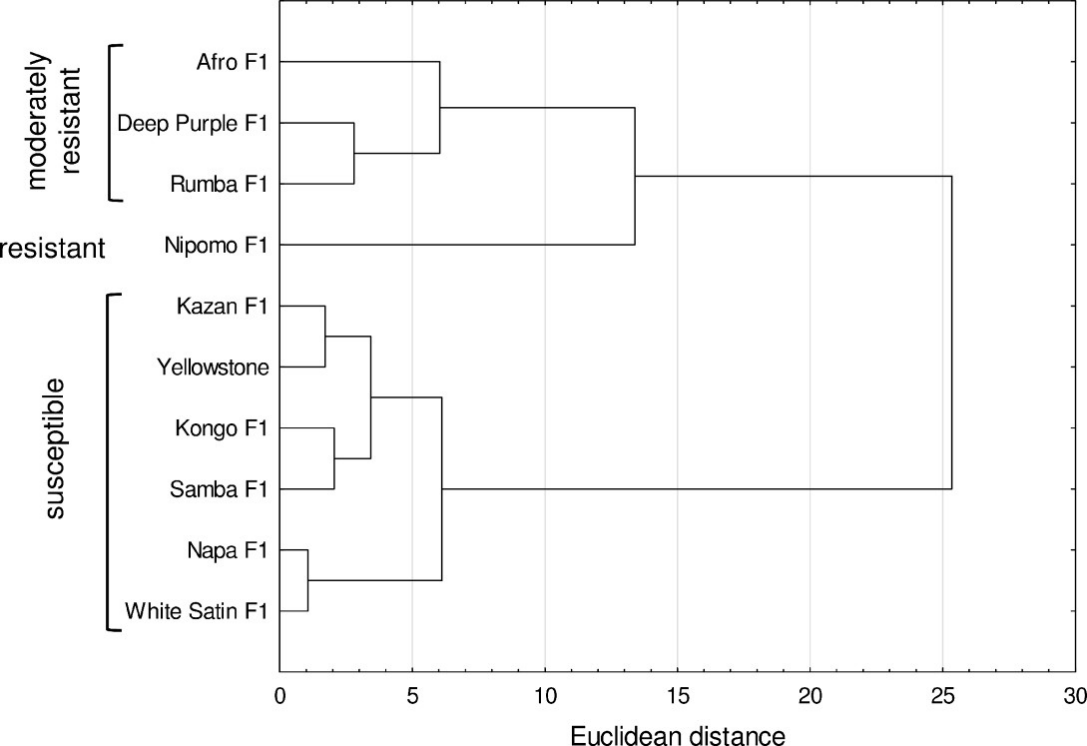
Dendrogram showing the clustering of carrot cultivars based on antibiosis data: Pre-reproductive period (*d*), reproductive period, fecundity of the female (*M*_*d*_), total progeny, intrinsic rate of increase (*r*_*m*_), mean generation time (*T*) and net reproduction rate (*R*_*o*_).

The dendrogram developed from the bionomic and demographic data for the hawthorn-carrot aphid populations revealed two clusters of cultivars characterized by different levels of antibiosis (Fig 4). The first cluster consisted of 4 cultivars, Afro F_1_, Deep Purple F_1_, and Rumba F_1_, it was characterized by moderate resistance. The cultivar Nipomo F_1_ showed the highest resistance which was inferred from the highest limitation of aphid development under laboratory conditions. The second cluster contained susceptible cultivars for which the data for demographic parameters indicated the existence of morphological and physiological characteristics favorable to the development of aphid populations (Fig 4).

Based on the calculated values of *r*_*m*_ and R_o_, Afro F_1_ and Nipomo F_1_ were classified as resistant, with Afro F_1_ having the highest level of antibiosis. Conversely, White Satin F_1_ and the other genotypes were classified as highly susceptible and susceptible, respectively (Table 2).

### Tolerance assessment

Two-way nested ANOVA showed significant differentiation at the level of cultivar and treatment (nested in cultivars) in terms of the analyzed features of the carrot root (Table 6).

**Table 6:** Two-way nested ANOVA for indicators (yield components) of the degree of tolerance to the hawthorn-carrot aphid, *Dysaphis crataegi*.

Yield components	Year	Source of variation			
		Cultivars		Treatment (Cultivars)	
		F-value	p-value	F-value	p-value
Root length [cm]	2011	3.065	0.006	2.122	0.045
	2012	1.246	0.295	0.751	0.673
Root weight [g]	2011	20.156	<0.001	7.697	<0.001
	2012	24.304	<0.001	26.958	<0.001
Sucrose content [mg/100 g FW]*	2011	43.342	<0.001	26.863	<0.001
	2012	45.340	<0.001	202.370	<0.001
Content of reducing sugars [mg/100 g FW]	2011	46.190	<0.001	174.860	<0.001
	2012	25.070	<0.001	475.190	<0.001
Content of carotenoids [mg/100 g FW]	2011	123.070	<0.001	11.124	<0.001
	2012	549.173	<0.001	12.459	<0.001
df	─	9		10	
Error df	─	40			

* FW = Fresh weight; significant at P<0.05.

**Fig 5:**
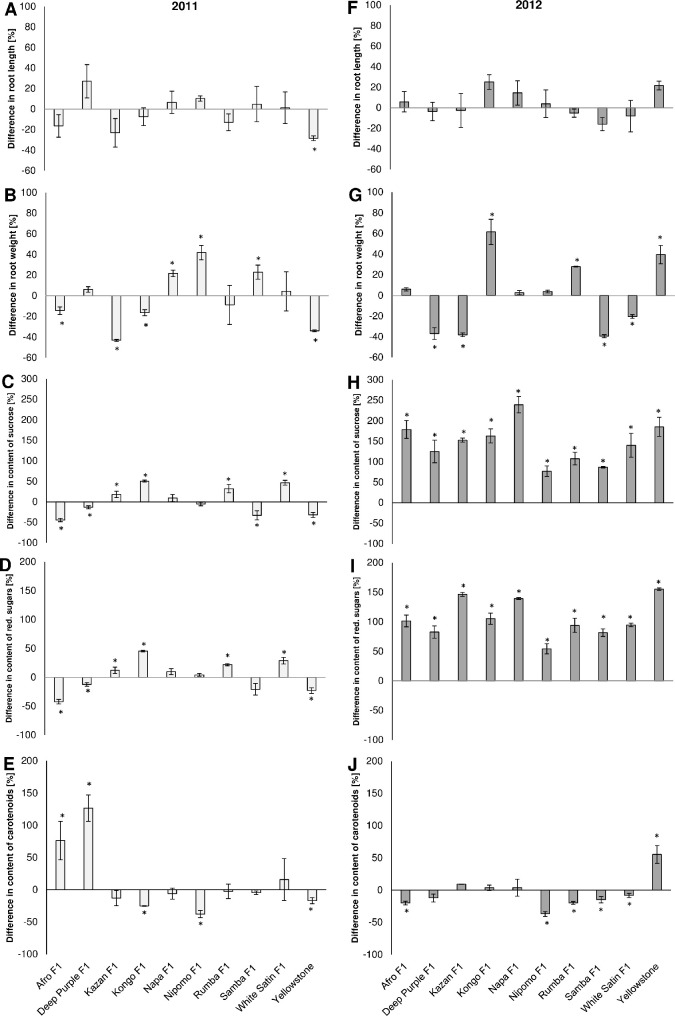
Effects of hawthorn-carrot aphid *(Dysaphis crataegi*) foraging on the length (A, F), weight (B, G), and contents of sucrose (C, H), reducing sugars (D, I) and carotenoids (E, J) in the roots of the ten tested carrot cultivars. * Significant differences between the control mean and the mean in colonized plants based on the Student’s t test conducted separately for each cultivar (P<0.05).

A statistically significant mean reduction (28.38%) of carrot roots length caused by feeding of the hawthorn-carrot aphid was registered only in the case of the cultivar Yellowstone in 2011, although there was a shortening of more than 20% in Kazan F_1_ or, conversely, a lengthening of Deep Purple F_1_ in 2011, and Kongo F_1_ and Yellowstone, in 2012 (Fig 5A and 5F).

Among the tested genotypes, only Kazan F_1_ responded to aphid feeding with a significantly lower mean root weight than the controls in both years (Fig 5B and 5G). In contrast, two genotypes, Nipomo F_1_ and Napa F_1_, had higher mean root weights (significantly higher for both in 2011, and for Nipomo F_1_ in 2012). In the case of the other cultivars, a different effect of hawthorn-carrot feeding aphid on the root weight was found. In 2011, significant decreases in weight were recorded for Afro F_1_, Kongo F_1_ and Yellowstone, while in 2012, significant weight decreases were recorded for Deep Purple F_1_, Samba F_1_ and White Satin F_1_ in comparison to the controls. The largest weight losses (approximately 40%) in comparison with to the control plants (on which the aphids did not feed) were recorded for Kazan F_1_ and Yellowstone in 2011, and Deep Purple F_1_, Kazan F_1_ and Samba F_1_ in 2012. In contrast, the root masses were significantly higher for Samba F_1_ in 2011 and for Kongo F_1_, Rumba F_1_ and Yellowstone in 2012 (Fig 5B and 5G).

In the roots of carrot plants infested by the hawthorn-carrot aphid, the contents of sucrose and reducing sugars were significantly higher than in the non-infested plants in four hybrid carrot cultivars, Kazan F_1_, Kongo F_1_, Rumba F_1_ and White Satin F_1_, in 2011, and in all tested cultivars in 2012 (Fig 5C, 5H, 5D and 5I). The highest increase in sucrose level (>200%) was recorded for Napa F_1_ in 2012 (Fig 5H). In addition, a high increase in sucrose concentration (>150%) was observed in the roots of Afro F_1_, Kazan F_1_, Kongo F_1_, and Yellowstone (Fig 5H). In 2011, Napa F_1_ and Nipomo F_1_, did not respond to the feeding of the hawthorn-carrot aphid with significant changes in their carbohydrate concentration (Fig 5C and 5D). In contrast, aphids caused significant reductions in the concentrations of sucrose and reducing sugars in the roots of Afro F_1_, Deep Purple F_1_, and Yellowstone, and a significant reduction in the level of sucrose in the roots of Samba F_1_ (Fig 5C and 5D).

In the tolerance experiment, there was significant increases in the contents of carotenoids only in the roots of Afro F_1_ and Deep Purple F_1_ in 2011, and in the roots of Yellowstone in 2012 (Fig 5E and 5J). In the case of Yellowstone and Afro F_1_, the opposite responses were was recorded in 2011 (Fig 5E). In addition, significant reductions in carotenoid level in the roots of Nipomo F_1_ in both years, in Kongo F_1_ in 2011 and in Rumba F_1_, Samba F_1_ and White Satin F_1_ in 2012, were recorded (Fig 5E and 5J).

Taking into account all the yield components assessed, the genotypes Kongo F_1_ and Napa F_1_ were considered to be highly tolerant to hawthorn-carrot aphid feeding. Moreover, Nipomo F_1_, Rumba F_1_ and White Satin F_1_ were categorized as having a moderate level of tolerance. The other cultivars were not tolerant, and among them, Afro F_1_, Deep Purple F_1_, Samba F_1_ and Yellowstone were considered to be the least tolerant (Table 2).

## Discussion

In the current study the abundance of hawthorn-carrot aphid in two consecutive years was mainly influenced by the carrot cultivar. Goszczyński and Cichocka [7] reported that the size of populations of hawthorn-carrot aphids on carrot were largely dependent on the number of migrants colonizing the plants and weather conditions in the spring and summer. In the present study, lower temperatures from the 3^rd^ week of June, a cold July, rainfall and many days with precipitation above 0.5 mm in 2011 may have restricted the migration and development of aphids on all cultivars in comparison to 2012. For instance, the cultivar Deep Purple F_1_ was non-infested (resistant) in 2011, but susceptible in 2012, when the weather conditions were more favorable to the development of the pest. Consequently, because of the weather, carrot cultivars that were understood to be resistant were not necessarily so. Thus, the screening and identification of resistant carrot cultivars in the field better reflects the real situation if the plants are exposed to a range of weather conditions.

Despite the inherent complication of interacting effects, field screening using naturally infested plants is effective in identifying genotypes that express resistance across a broad range of growing conditions. In the field experiment, a group of five genotypes, Afro F1, Nipomo F1, Samba F1, White Satin F1 and Yellowstone, showed moderate resistance to aphid feeding and development which was reflected in both low aphid populations and a low percentage of infested plants in both growing seasons. Two cultivars, Deep Purple F1 and Yellowstone, were moderately resistant, and one cultivar, White Satin F1, was resistant to alate colonizers. This level of resistance was demonstrated by the low numbers of migrating alates colonizing these cultivars. However, it is difficult to ascertain which of the mechanisms, antixenosis and/or antibiosis, contributed most to the difference in the level of field resistance to the pest because the experiment involved both mechanisms. Antixenosis testing is essentially based on measuring the attractiveness of a plant genotype to alate aphids, but unfortunately, the selection process can be disrupted under such conditions [48]. Aphids are relatively weak flyers, being able to determine their own speed and direction only at low wind speeds. As a consequence, only a very small proportion of alates locate suitable hosts [49]. In our study, the weak colonization by alates and low aphid abundance on Deep Purple F_1_, White Satin F_1_ and Yellowstone in two consecutive seasons may indicate host plant selection and the antixenosis (no preference) mechanism of resistance. Antixenosis has been documented in horticultural brassicas, wheat and sugar beet for the cabbage aphid, *Brevicoryne brassicae* L., English grain aphid, *Sitobion avenae* (Fabr.) and black bean aphid, *Aphis fabae* Scop., respectively [50–52]. However, to confirm this mechanism of resistance to plant colonization by migrating alates, a choice test should be performed under controlled conditions [16].

The decision on the suitability of the plant as a host is made in the very first phase of host selection, with alate colonizers using both visual and chemical cues [49, 53, 54]. Several factors can affect the selection of the host plant including the physical properties of plants (color and morphological characteristics), emission of volatiles, metabolic profiles of the plant, and environmental conditions [55–57]. Aphids might show a preference for a specific odor, hue or intensity of color in their preferred plant [58–60].

In the present study, the cultivars resistant to infestation by winged migrant aphids, Deep Purple F_1_, White Satin F_1_ and Yellowstone, stood out among other varieties due to their very light (pale) leaf color (White Satin F_1_ and Yellowstone) and purple hue (Deep Purple F_1_), while susceptible cultivars had intense green leaves (unpublished data). Nazeeret et al. [61] investigated the level of resistance of seven Chinese cabbage cultivars to the green peach aphid, *Myzus persicae* (Sulz.) and found that aphids preferred cultivars with the longest wavelengths of leaf color. In addition, direct evidence of green color preference by the bird cherry-oat aphid, *Rhopalosiphum padi* L on bird cherry, was reported by Archetti and Leather [62]. However, aphids usually prefer the color yellow [63]. Łuczak [52] reported that the lack of colonization of single-sprout varieties of sugar beet by the black bean aphid is due to the low concentrations of flavonoids and carotenoids. The color preferences of the hawthorn-carrot aphid appear to be similar to that of the green peach aphid for Chinese cabbage cultivars, however, to confirm this hypothesis, separate studies on the behavioral response of the hawthorn-carrot aphid to colors are necessary.

Immediately after landing, aphids perform a preliminary assessment of the plant, during which they examine its surface [64]. Different morphological characteristics such as trichomes and hardness or thickness of leaf tissues can play important roles in herbivore preference [54, 65]. Trichome density has a significant influence on aphid feeding by affecting aphid movement and stylet insertion. In addition, the glandular trichomes produce toxic exudates that trap aphids and kill them [66]. We explored one of the mechanisms that might be responsible for attracting or repelling hawthorn-carrot aphids by testing the length, thickness, and density of trichomes on the leaves and leaf petioles of the tested carrot cultivars. In our experiments, the highest density was 71.94 trichomes/cm^2^ on the leaf petioles of Nipomo F_1_ which exhibited moderate field resistance and high levels of antibiosis. By contrast, the lowest density was from 0.00 to 2.20 trichomes/cm^2^ on the leaf petioles of the cultivars White Satin F_1_ and Yellowstone with high and moderate field resistance, respectively, but very low degrees of antibiosis. Our results suggest that a very high density of trichomes on the leaf petioles of the cultivar Nipomo F_1_ could adversely affect the feeding and demographic parameters of the aphid. The negative, although not significant, values of the correlation (from r = -0.373 to r = -0.548) between the density of trichomes on leaf petioles and the demographic parameters of the hawthorn-carrot aphid indicate such a tendency (S2 Table). Therefore, we strongly recommend the use of genotypes with dense hairs on the leaf petioles in a future study to verify the importance of this mechanism. Having stated that, there is some uncertainty regarding the influence of trichomes on host selection by aphids. It has been reported that trichomes did not affect the selection of host or the biology of the cotton aphid, *Aphis gossypii* Glöver on cotton [67], and the cowpea aphid, *Aphis craccivora* C.L. Koch on cowpea [68], and on lima bean [69]. However, leaf trichomes can mechanically disrupt the movement of insect herbivores on the leaf surface, thus reducing access to the food. Leaf hairiness was partly responsible for the poorer performance of cotton aphid on some cotton and okra varieties [70, 71]. For this reason, the hairy cultivars were used for their higher resistance against various insects on cotton, wheat, and soybean [53].

The low values of aphid abundance indicators (mean number of aphids throughout the growing season, mean number of aphids and mean percentage of infested plants at maximum abundance) obtained from field tests on the cultivars, Afro F_1_, Nipomo F_1_, Samba F_1_, White Satin F_1_, and Yellowstone indicate that the hawthorn-carrot aphid was not able to successfully build up large populations on the plants under natural conditions, which may have resulted from antixenosis during probing and/or antibiosis during feeding [11, 66].

The hawthorn-carrot aphid feeds on the bases of the leaf petioles and root neck before moving towards the root. Therefore, their nutrients concentrations may be important in the process of settlement by aphids. Plant chemical composition is a crucial determinant of host plant–insect interactions. In plant tissues, there are approximately 200,000 metabolites, and the number in each plant species is approximately 15,000 [72]. The most important nutrients for aphids are nitrogen compounds, such as free amino acids, low molecular weight amides, peptides, nucleotides, and proteins [73, 74]. However, sugars are one of the principal determiners of the acceptability of a plant as a food source for many insect species [74, 75]. High concentrations of water soluble carbohydrates (WSC) in plant tissues often reduce aphid performance because they need to secrete these compounds as honey dew to maintain osmotic neutrality [76, 77]. Alkhedir et al. [78] stated that high WSC levels are responsible for the resistance of cocksfoot grass cultivars to the English green aphid. In our experiment, the soluble sugar and sucrose contents in the root neck apexes and leaf petioles were the highest in the cultivars White Satin F_1_ and Yellowstone (S3 Table), which had high and moderate field resistance, respectively. However, the laboratory study of aphid development on these two cultivars contradicted the results of the field evaluation of aphid performance because aphid reproduction under laboratory conditions was very high on both. The concentration of water-soluble carbohydrates in the phloem sap depended on the environmental conditions of the plant species and the developmental stage [79, 80]. In the present study, the sugar concentrations in the leaf petioles and root neck apex of the tested carrot cultivars were determined when the plants were 11 weeks old, whereas the laboratory tests were performed on younger plants (7 weeks old). These results suggest that more specific tests need to be performed with whole plants, such as studies of the concentrations of nutrient and volatile compounds, and other possible causes of resistance.

Understanding the demographic parameters of a pest is essential to the development of an integrated pest management strategy. These parameters define the potential for the population growth of an insect pest in the current and following generation. Life table parameters, particularly the intrinsic rate of increase, are the most important parameters that can be used to assess a plant’s level of resistance (antibiosis) to insects [16, 81, 82]. In our experiments, the fecundity, reproductive period, intrinsic rate of population growth and net reproductive rate were significantly lower for the resistant cultivars, Nipomo F_1_ and Afro F_1_ in comparison to the highly susceptible cultivar, White Satin F_1_. The reduction in the reproductive performance of the hawthorn-carrot aphid on the resistant cultivars suggests that antibiosis may be the modality of resistance. Nipomo F_1_ and Afro F_1_ are therefore promising cultivars for carrot breeding programs aimed at developing cultivars resistant to hawthorn-carrot aphid. The expression of antibiosis in these genotypes may arise from the presence of primary and/or secondary plant metabolites, growth inhibition, reduced levels of nutrients, or the presence of inhibitors, or various combinations of these. Further detailed studies on the determination of biochemical and morphological characteristics that induce resistance need to be carried out.

Tolerance is also an important characteristic of the resistance of carrot cultivars to the potential impacts of hawthorn-carrot aphid infestation. The evaluation of carrot tolerance in our experiment was difficult and time-consuming, with inconsistent results generated in successive years in the field experiments. Only one cultivar, Kazan F_1_, responded to aphid feeding with significantly lower root weight in both years, while Napa F_1_ and Nipomo F_1_ responded oppositely, i.e., with higher weight in both years, but significantly higher only in 2011. A different response to hawthorn-carrot aphid foraging in the same cultivar in subsequent years could have resulted from markedly different weather conditions that had a direct impact on the aphids’ feeding and host-plant quality [83, 84]. In susceptible cultivars, even moderately high populations of the sugar beet root aphid, *Pemphigus betae* Doane induced significant reductions in sugar beet yield, and sugar and recoverable sugar levels [85]. However, several sugar beet genotypes showed tolerance to beet root aphid [86]. Reductions of the root mass of carrot and potato plants due to the feeding of black bean aphid and green peach aphid, respectively, were reported by Łuczak et al. [87] and Hoysted et al. [88], respectively. Łuczak [52] further reported that black bean aphid did not always cause a decrease in the yield of sugar beet roots, which depended largely on the size of the aphid colonies. In tolerant cultivars, an increase in root mass was noted despite the feeding of large aphid colonies.

In the current study the pest aphid had different effects on the nutrients levels of the roots of different carrot cultivars. Aphids significantly increased the sugar content in the roots of all the tested carrot cultivars in 2012, but in 2011, the same effect was observed only in four genotypes, namely Kazan F_1_, Kongo F_1_, Rumba F_1_ and White Satin F_1_. However, there were significant reductions in the concentrations of both sucrose and reducing sugars in the roots of Afro F_1_, Deep Purple F_1_, and Yellowstone. Łuczak et al. [87] reported that as a consequence of the feeding of black bean aphid on carrot leaves, the contents of sucrose and reducing sugars in the roots, depending on the variety, varied by more than a 100% increase to a decrease of 28.6%. In another study, decreases in yield and the levels of sugars were recorded in sugar beet cultivars susceptible to the foraging of the black bean aphid [52]. It was also reported that the feeding of the English grain aphid on tolerant genotypes induced an increase in the content of soluble sugars in the ears of winter wheat, while in susceptible varieties, the amounts of these components decreased [89], and feeding of the rosy apple aphid, *Dysaphis plantaginea* Pass. on apple shoots increased the concentration of reducing sugars [90]. Based on the results reported in these four papers, aphids’ salivary components may disrupt normal plant physiology which manifests as growth reduction and altered biochemical composition. In the present body of research we observed that the hawthorn-carrot aphid produces large amounts of honeydew during feeding on the root neck and carrot roots. The composition of honeydew varies among species, but it mainly contains glucose, sucrose, fructose and melezitose [91]. The increase in the concentrations of sugars in the roots of some tested carrot cultivars may have been caused by the diffusion of sugars contained in honeydew into the roots; however, this suggestion needs to be confirmed.

Stress induced by aphid feeding can speed up tissue aging and may cause higher carotenoid content [92]. In our study, a significant increase in carotenoid content was observed only in the roots of Afro F_1_ and Deep Purple F_1_ in 2011. A very high percentage increase of total carotenoid concentration in the roots of Yellowstone resulted from the low level of accumulation of these pigments in roots of this cultivar (S3 Table). In contrast, only Nipomo F_1_ in 2011 and 2012 and Kongo F_1_ in 2011 registered significant decreases in the carotenoid content. Heng-Moss et al. [35] and Ni et al. [93] documented reductions in the total carotenoid levels in response to the feeding of the Russian wheat aphid, *Diuraphis noxia* Kurd. on the damaged regions of wheat leaves. In addition, triticale genotypes showed a similar response to feeding by the English grain aphid [94]. Given the existing uncertainties, the mechanisms that underlie carrot plant tolerance to hawthorn-carrot aphid require explanation in future research.

In this research, the tested carrot cultivars had a range of responses to hawthorn-carrot aphid feeding across a number of morphological and biochemical variables. Therefore, it can be concluded that these responses were manifestations of genetic differences amongst the cultivars. Overall, the hawthorn-carrot aphid was best adapted to exploit Kazan F_1_ because it was the most attractive variety, which was evidenced by a high number of migrants and the most palatable variety, as demonstrated by the highest number of feeding aphids throughout the season and a high finite rate of increase. Furthermore, the moderate level of field resistance of Afro F_1_ and Nipomo F_1_ was most likely attributable to antibiosis, as evidenced by the low intrinsic rate of increase (r_m_). That said, field resistance in the moderately tolerant genotype, White Satin F_1_, was not confirmed in antibiosis experiments. The low number of aphids probably resulted from the low attractiveness of these genotypes to migrants which might have reflected antixenosis. Therefore, to investigate the potential involvement of this mechanism, we recommend a laboratory experiment in which alate aphids are allowed to freely choose among the tested carrot genotypes.

The high levels of tolerance of the two susceptible carrot genotypes, Kongo F_1_ and Napa F_1_, seen in the high intrinsic rate of increase (r_m_) (low level of antibiosis), indicate that these cultivars are able to compensate for, or tolerate, aphid foraging. Also, the very low r_m_ of Nipomo F_1_ suggests that moderate tolerance in this cultivar could be a consequence of a high level of antibiosis, which could limit the development of aphids on the infested plants. Thus, the genotypes Afro F_1_, Kongo F_1_, Napa F_1_ and Nipomo F_1_ should be considered for use in future studies as sources of resistance and/or tolerance genes.

In conclusion, we detected substantial differences between years for the preference, performance and reaction of the same genotypes to aphids and their feeding. Therefore, we recommend a combination of laboratory and long-term field experiments in carrot growing-regions to identify cultivars/lines that consistently show high resistance to hawthorn-carrot aphid infestation.

## Supporting information

S1 Table Rainfall and average daily temperature at the experimental site (Mydlniki, Krakow region, Poland) in the seasons 2011 and 2012. (DOCX)

S2 Table Coefficients of correlation *(r)* between the abundance of *Dysaphis crataegi* and density of trichomes on tested carrot cultivars, N = 10. (DOCX)

S3 Table Differentiation of traits of the roots of the control plants of the tested carrot cultivars. Means within a column followed by the same letter(s) are not significantly different (Duncan’ Multiple Range Test, p<0.05).
(DOCX)
