# Supplementary material for: Resistance and tolerance of ten carrot cultivars to the hawthorn-carrot aphid, Dysaphis crataegi Kalt., in Poland
Source: PLoS One. 2021 Mar 2;16(3):e0247978. doi: 10.1371/journal.pone.0247978 (PMC7924882; doi:10.1371/journal.pone.0247978)
Supplement: S1 Table — (DOCX) [file pone.0247978.s001.docx]

**S1 Table. Rainfall and average daily temperature at the experimental site (Mydlniki, Krakow region, Poland in the seasons 2011 and 2012.**

| **Year/month/decade** | **Weather parameter** | **Average daily**  **temperature (°C)** | | **Sum of**  **precipitation (mm)** | | **No. of days with precipitation ≥ 0.05 mm** | |
| --- | --- | --- | --- | --- | --- | --- | --- |
|  |  | 2011 | 2012 | 2011 | 2012 | 2011 | 2012 |
| May | 1 decade | 10.8 | 13.3 | 16.3 | 9.9 | 4 | 2 |
|  | 2 decade | 15.7 | 12.0 | 13.9 | 6.6 | 3 | 2 |
|  | 3 decade | 15.8 | 17.1 | 24.4 | 6.9 | 3 | 2 |
| June | 1 decade | 19.2 | 17.3 | 2.9 | 87.5 | 2 | 6 |
|  | 2 decade | 18.4 | 18.9 | 11.4 | 21.5 | 3 | 4 |
|  | 3 decade | 17.9 | 19.3 | 26.0 | 5.0 | 4 | 1 |
| July | 1 decade | 17.3 | 23.5 | 44.7 | 10.7 | 6 | 4 |
|  | 2 decade | 19.1 | 19.2 | 62.6 | 31.7 | 6 | 4 |
|  | 3 decade | 16.5 | 21.5 | 55.5 | 3.6 | 8 | 2 |
| August | 1 decade | 19.5 | 20.6 | 12.3 | 24.7 | 3 | 3 |
|  | 2 decade | 19.0 | 20.8 | 20.1 | 11.6 | 4 | 4 |
|  | 3 decade | 19.7 | 19.5 | 5.0 | 4.3 | 3 | 3 |
| September | 1 decade | 16.2 | 17.9 | 9.1 | 5.4 | 2 | 3 |
|  | 2 decade | 15.3 | 16.1 | 1.6 | 11.6 | 1 | 3 |
|  | 3 decade | 13.2 | 14.6 | 7.5 | 15.8 | 2 | 3 |
