# Supplementary material for: Resistance and tolerance of ten carrot cultivars to the hawthorn-carrot aphid, Dysaphis crataegi Kalt., in Poland
Source: PLoS One. 2021 Mar 2;16(3):e0247978. doi: 10.1371/journal.pone.0247978 (PMC7924882; doi:10.1371/journal.pone.0247978)
Supplement: S2 Table — (DOCX) [file pone.0247978.s002.docx]

**S2 Table. Coefficients of correlation *(r)* between the abundance and performance of *Dysaphis crataegi* and density of trichomes on tested carrot cultivars in 2011-2012, N=10**

| **Parameters** | **Mean number / 1 cm^2^ of leaf petiole** | | |
| --- | --- | --- | --- |
|  | Year | r | *P* |
| Mean number of migrants | 2011 | -0.135 | 0.477 |
|  | 2012 | -0.556 | 0.770 |
| Mean % of plants colonized by migrants | 2011 | -0.131 | 0.491 |
|  | 2012 | 0.005 | 0.979 |
| Mean seasonal number of aphids | 2011 | -0.1859 | 0.325 |
|  | 2012 | 0.182 | 0.924 |
| Mean number of aphids in peak aphid abundance | 2011 | -0.251 | 0.180 |
|  | 2012 | -0.153 | 0.420 |
| Mean % of infested plants in peak aphid abundance | 2011 | 0,184 | 0.923 |
|  | 2012 | -0.934 | 0.623 |
| *R_o_* net reproductive rate | 2012* | -0.548 | 0.101 |
| *T* mean generation time |  | -0.419 | 0.228 |

* The experiment was carried out under laboratory conditions in 2012.
