# Supplementary material for: Resistance and tolerance of ten carrot cultivars to the hawthorn-carrot aphid, Dysaphis crataegi Kalt., in Poland
Source: PLoS One. 2021 Mar 2;16(3):e0247978. doi: 10.1371/journal.pone.0247978 (PMC7924882; doi:10.1371/journal.pone.0247978)
Supplement: S3 Table — Means within a column followed by the same letter(s) are not significantly different (Duncan’ Multiple Range Test, p<0.05). (DOCX) [file pone.0247978.s003.docx]

**S3 Table. Differentiation of traits of the roots of the control plants of the tested carrot cultivars (one-way ANOVA, factor df = 9, error df = 20).**

| **Cultivar/Year** | **Mean length of 1 root**  **(± SE) [cm]** | **Mean weight of**  **1 root (± SE) [g]** | **Mean quantity (± SE) [mg/100 g FW*]** | | | **Mean length of 1 root**  **(± SE) [cm]** | **Mean weight /**  **1 root (± SE) [g]** | **Mean quantity (± SE) [mg /100 g FW]** | | |
| --- | --- | --- | --- | --- | --- | --- | --- | --- | --- | --- |
|  |  |  | **reducing sugars** | **sucrose** | **carotenoids** |  |  | **reducing sugars** | **sucrose** | **carotenoids** |
|  | **2011** | | | | | **2012** | | | | |
| Afro F_1_ | 11.5±0.6 bc | 56.4±1.3 a | 25.14±0.80 ab | 22.38±0.78 a | 6.78±1.11 d | 8.04±0.99 a | 26.0±0.5 bc | 16.86±0.72 bc | 10.36±0.79 cde | 8.40±0.30 d |
| Deep Purple F_1_ | 10.4±1.5 c | 34.2±2.1 de | 18.37±0.35 cde | 16.36±0.37 bc | 2.56±0.02 e | 9.96±0.69 a | 22.0±2.1 c | 9.16±0.38 e | 6.62±0.35 f | 0.58±0.04 e |
| Kazan F_1_ | 14.8±1.5 a | 55.1±1.4 a | 14.68±0.37 fg | 11.75±0.43 d | 14.23±1.33 b | 8.13±1.69 a | 28.8±0.6 b | 13.74±0.25 d | 11.98±0.26 bcd | 17.90±0.52 a |
| Kongo F_1_ | 11.4±0.6 bc | 58.3±0.9 a | 19.41±0.33 cd | 18.39±0.46 b | 16.53±0.82 a | 8.32±0.22 a | 22.7±1.2 c | 13.08±0.47 d | 9.33±0.34 e | 13.11±0.02 b |
| Napa F_1_ | 11.2±0.7 bc | 37.8±1.2 cd | 17.86±0.35 de | 13.93±0.50 cd | 8.01±0.01 cd | 8.50±1.83 a | 26.2±2.6 bc | 17.84±0.18 b | 10.96±0.71 bcde | 9.21±0.43 d |
| Nipomo F_1_ | 10.0±0.4 c | 39.1±2.3 c | 16.26±0.19 ef | 14.24±0.37 cd | 12.42±1.06 b | 7.56±0.86 a | 23.5±0.5 c | 17.90±0.67 b | 13.20±0.62 b | 12.56±0.28 bc |
| Rumba F_1_ | 9.2±0.3 c | 31.9±1.8 e | 12.16±0.41 g | 8.40±0.41 e | 8.79±0.61 cd | 8.41±1.28 a | 22.1±1.1 c | 15.48±0.55 c | 12.19±0.48 bc | 8.48±0.36 d |
| Samba F_1_ | 9.3±1.2 c | 32.0±1.5 e | 27.24±2.62 a | 23.09±2.84 a | 9.41±0.24 c | 9.16±0.61 a | 39.2±1.2 a | 21.81±0.43 a | 17.04±0.30 a | 11.88±0.12 c |
| White Satin F_1_ | 11.6±0.3 bc | 58.0±1.2 a | 21.11±0.59 c | 15.87±0.41 bc | 0.15±0.04 f | 9.04±0.42 a | 28.4±0.4 b | 16.71±0.43 bc | 11.22±1.85 bcde | 0.20±0.01 e |
| Yellowstone | 13.6±0.2 ab | 44.4±0.6 b | 23.88±0.36 b | 22.49±0.35 a | 1.94±0.02 ef | 8.46±0.15 a | 16.8±0.9 d | 12.34±0.17 d | 9.67±0.32 de | 0.48±0.04 e |
| F-value | 4.061 | 54.250 | 26.265 | 24.024 | 56.267 | 0.428 | 20.837 | 59.08 | 13.204 | 476.156 |
| p-value | 0.004 | <0.000 | <0.000 | <0.000 | <0.000 | 0.904 | <0.000 | <0.000 | <0.000 | <0.000 |

* FW=Fresh weight.

Means within a column followed by the same letter(s) are not significantly different (Duncan’s Multiple Range Test p < 0.05)
